# Sudden cardiac arrest in a 47-year-old female with hypertrophic obstructive cardiomyopathy and anomalous papillary muscle insertion: a case report with advanced multimodal imaging, pathophysiological insights, and evidence-based surgical management

**DOI:** 10.1093/ehjcr/ytaf616

**Published:** 2025-11-26

**Authors:** Burak Oezdemir, Moritz Seiffert, Matthias Bechtel, Justus Strauch, Aydan Ewers

**Affiliations:** Department of Cardiology and Angiology (Medical Clinic II), University Hospital Bergmannsheil, Ruhr University Bochum, Bürkle-de-la-Camp-Platz 1, Bochum 44789, Germany; Department of Cardiology and Angiology (Medical Clinic II), University Hospital Bergmannsheil, Ruhr University Bochum, Bürkle-de-la-Camp-Platz 1, Bochum 44789, Germany; Department of Cardiac and Thoracic Surgery, University Hospital Bergmannsheil, Ruhr University Bochum, Bürkle-de-la-Camp-Platz 1, Bochum 44789, Germany; Department of Cardiac and Thoracic Surgery, University Hospital Bergmannsheil, Ruhr University Bochum, Bürkle-de-la-Camp-Platz 1, Bochum 44789, Germany; Department of Cardiology and Angiology (Medical Clinic II), University Hospital Bergmannsheil, Ruhr University Bochum, Bürkle-de-la-Camp-Platz 1, Bochum 44789, Germany

**Keywords:** Hypertrophic obstructive cardiomyopathy, Anomalous papillary muscle insertion, Sudden cardiac arrest, Subvalvular obstruction, Septal myectomy, Multimodality imaging, Ventricular fibrillation, Case report

## Abstract

**Background:**

Hypertrophic obstructive cardiomyopathy (HOCM) with anomalous papillary muscle insertion (APMI) is a rare morphological variant that can cause fixed subvalvular obstruction and malignant arrhythmias. Diagnostic assessment becomes especially challenging when significant gradients occur without systolic anterior motion (SAM), making multimodal imaging crucial.

**Case summary:**

A 47-year-old woman experienced out-of-hospital cardiac arrest due to ventricular fibrillation during physical exertion. Transthoracic and transesophageal echocardiography revealed a hypertrophied anterolateral papillary muscle inserting directly into the anterior mitral leaflet at the aortomitral junction, forming a rigid subvalvular ridge without SAM. Cardiac magnetic resonance showed concentric hypertrophy and preserved function without fibrosis. Extended septal myectomy and resection of the anomalous papillary muscle resulted in complete resolution of obstruction. Postoperative recovery was uneventful, and the patient remains under structured follow-up.

**Discussion:**

This case illustrates how multimodality imaging is crucial for recognizing uncommon subvalvular variants of hypertrophic obstructive cardiomyopathy and for guiding optimal therapeutic decision-making. Surgical success in such patients relies on complete resection of obstructive structures. While ICD implantation was considered according to secondary prevention guidelines, the decision was deferred after successful surgical correction and interim protection with a wearable defibrillator. Conventional HCM risk models may not fully capture the risk profile of these rare phenotypes, underscoring the importance of individualized management.

Learning points

- Anomalous papillary muscle insertion (APMI) represents an underrecognized morphological variant of hypertrophic obstructive cardiomyopathy (HOCM) that can cause fixed subvalvular obstruction and precipitate malignant arrhythmias or sudden cardiac arrest, even in the absence of classical systolic anterior motion (SAM).
- Multimodality imaging—particularly transesophageal echocardiography and cardiac magnetic resonance—plays a pivotal role in identifying APMI, characterizing its anatomical extent, and guiding tailored surgical strategies beyond standard septal myectomy.

## Introduction

Hypertrophic cardiomyopathy (HCM) is the most common inherited cardiac disorder and an important cause of sudden cardiac death (SCD). While systolic anterior motion (SAM) of the mitral valve is the classical mechanism of obstruction, subvalvular variants such as anomalous papillary muscle insertion (APMI) represent distinct and clinically significant phenotypes. Klues *et al*.^1^ reported direct papillary muscle insertion into the anterior mitral leaflet in ∼13% of excised valves in obstructive HCM. Advanced multimodality imaging is essential for accurate diagnosis and surgical planning.

## Summary figure

**Figure:**
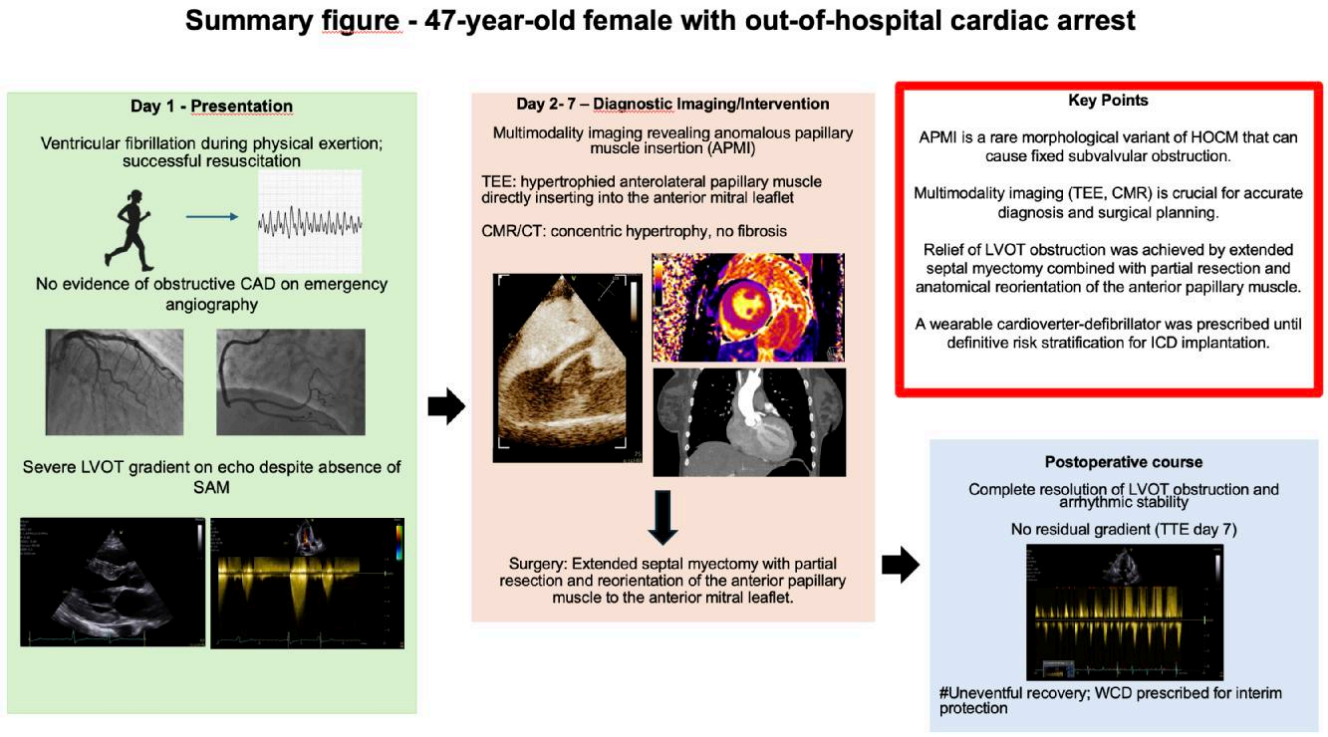


## Case presentation

A 47-year-old woman (BSA 1.7 m^2^) without prior cardiac history collapsed while running. Bystander CPR was initiated; emergency services documented ventricular fibrillation. Return of spontaneous circulation was achieved after two shocks. ECG showed LV hypertrophy and non-specific repolarization changes. Coronary angiography excluded obstructive disease. CT ruled out aortic dissection, pulmonary embolism, and cerebral hemorrhage but revealed small embolic infarcts. The patient was extubated the next day.

**Figure 1:**
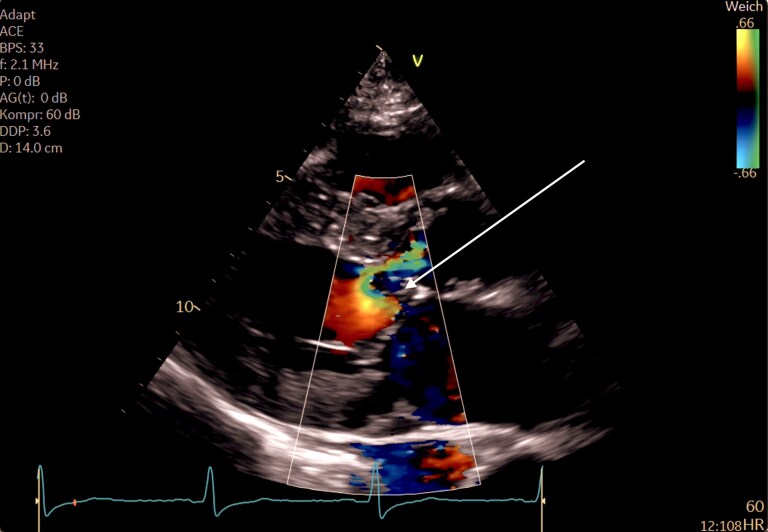
Color Doppler imaging demonstrates pronounced aliasing and flow acceleration within the left ventricular outflow tract, indicating dynamic obstruction without evidence of systolic anterior motion (see Supplementary material online, TTE  *Videos S1–S3*).

**Figure 2:**
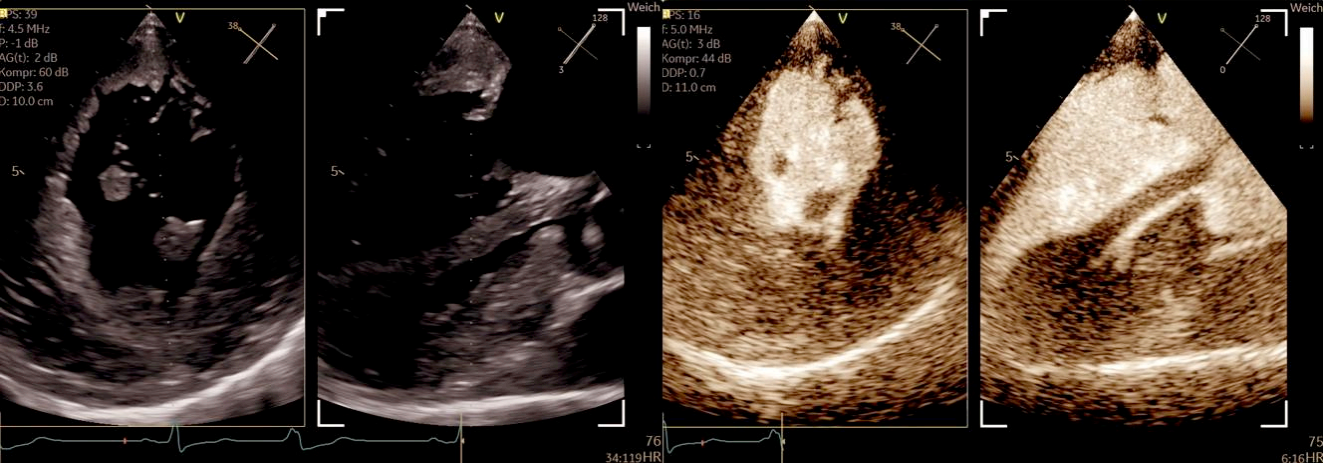
Multiplanar transgastric TEE views (long- and short-axis) after contrast administration. The images demonstrate pronounced concentric left ventricular hypertrophy with markedly thickened papillary muscles. After contrast enhancement, the anatomical details are more clearly visualized, including the insertion of the hypertrophied anterolateral papillary muscle into the region of the aortomitral continuity (see Supplementary material online, TTE  *Videos S1–S4*).

**Figure 3:**
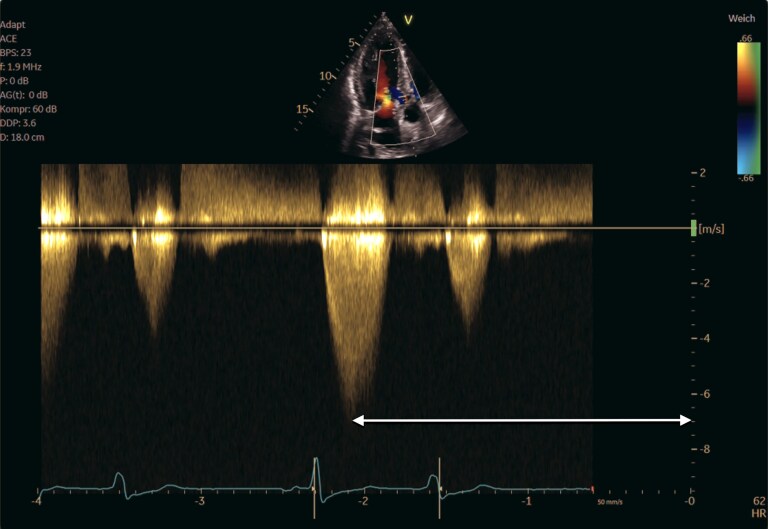
Continuous-wave Doppler assessment across the left ventricular outflow tract. The Doppler spectrum demonstrates a markedly elevated peak velocity (Vmax 7.3 m/s), corresponding to a resting peak gradient of 212 mmHg and a mean gradient of 105 mmHg. These findings indicate severe subvalvular obstruction due to a rigid, hypertrophied muscular ridge within the LVOT.

Transthoracic echocardiography (TTE) revealed preserved systolic function and concentric hypertrophy (septal thickness 13 mm) with markedly elevated LVOT gradients (*Figure 1*). Transesophageal echocardiography (TOE) demonstrated a hypertrophied anterolateral papillary muscle inserting directly into the aortomitral junction, forming a rigid subvalvular ridge and causing fixed LVOT obstruction (*Figure 2*), without the evidence of SAM. Peak gradients were 212 mmHg (mean 105 mmHg, Vmax 7.3 m/s)—(*Figure 3*). Mild central aortic regurgitation was also observed.

**Figure 4:**
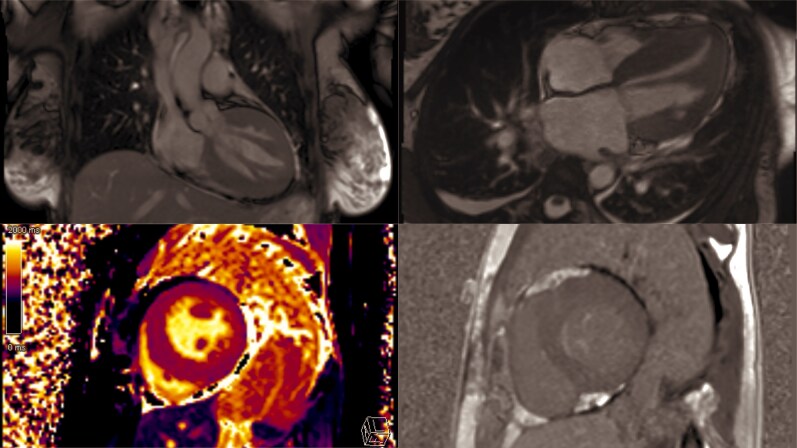
Multiplanar cardiac MRI assessment demonstrating left ventricular morphology, function, and tissue characteristics.

**Figure 5:**
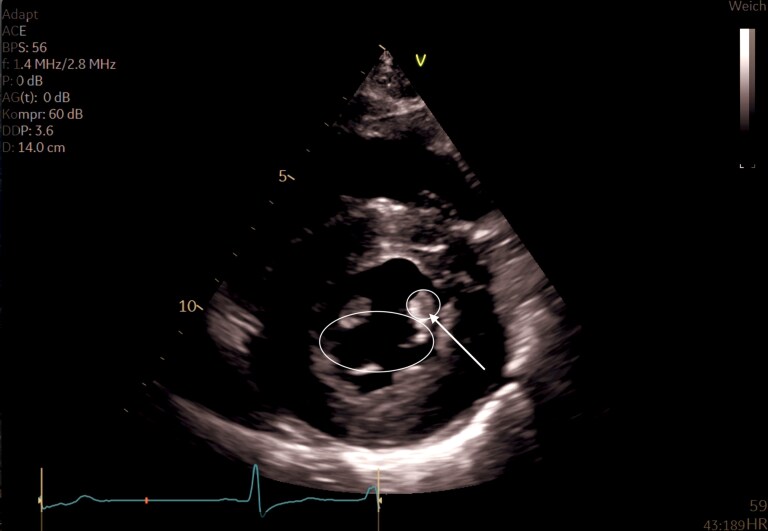
Parasternal short-axis view demonstrating the ‘lip-nevus sign.’ The mitral valve leaflets appear as opposing ‘lips,’ while a markedly hypertrophied anterolateral papillary muscle (arrow) is already visible at the level of the mitral valve, resembling a central ‘nevus.’ This finding strongly suggests anomalous papillary muscle insertion.

Cardiac magnetic resonance confirmed concentric hypertrophy and preserved systolic function without fibrosis (*Figure 4*). Echocardiographic imaging also revealed features consistent with the ‘lip-nevus sign’, a recently described marker facilitating recognition of APMI, which was demonstrated in *Figure 5*.

The patient was stabilized on beta-blockers, aspirin, and statins, and discharged with a wearable cardioverter-defibrillator. After multidisciplinary evaluation, surgery was performed via a transaortic approach: Extended septal myectomy and targeted resection of the anomalous papillary muscle. Intraoperative TOE confirmed resolution of obstruction and restoration of normal mitral valve motion.

**Figure 6:**
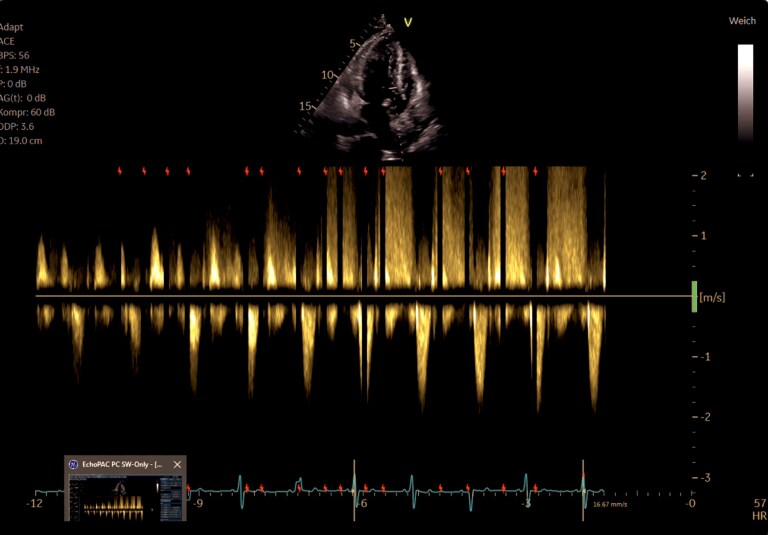
Continuous-wave Doppler across the left ventricular outflow tract postoperatively. Postoperative TTE shows no detectable pressure gradient across the LVOT. Flow velocities remain within physiological range, indicating complete resolution of dynamic or fixed subvalvular obstruction (see Supplementary material online, postoperative TTE  *Videos S1* and *S2*).

The postoperative course was uneventful. On postoperative day 7, TTE confirmed complete resolution of LVOT obstruction with no residual gradient (*Figure 6*). The patient was discharged on day 10 and remains under follow-up for ongoing evaluation of ICD implantation. Genetic testing has been arranged for the first outpatient visit; cascade screening of first-degree relatives will be offered based on the results.

## Discussion

This case underscores the pivotal role of multimodality imaging in detecting uncommon subvalvular variants of hypertrophic obstructive cardiomyopathy (HOCM), which is essential for planning an optimal therapeutic strategy. Accurate recognition of such anatomical variants enables tailored surgical approaches and prevents residual obstruction.

APMI is a well-recognized but underreported morphological variant in obstructive HCM. Klues *et al*.^1^ first described direct insertion of papillary muscles into the anterior mitral leaflet in ∼13% of excised valves from HOCM patients.^1^ Such abnormal attachments can produce fixed obstruction, increased shear stress, and heightened arrhythmic propensity. In our patient, these mechanisms likely contributed to the malignant arrhythmic event.

Surgical treatment in this subgroup requires particular attention. Standard extended septal myectomy, although effective in most patients, may fail if anomalous subvalvular structures remain intact. Carvalho *et al*.^2^ demonstrated the necessity of combined septal and subvalvular resection in patients with APMI, reporting excellent hemodynamic outcomes.^2^ Similarly, Mutsuga *et al*.^3^ showed that residual gradients persisted in cases where subvalvular anomalies were not addressed, reinforcing the importance of comprehensive resection.^3^ Recent series, including that of Fernández *et al*.^4^ confirmed that tailored surgery in APMI-positive patients results in favorable outcomes.^4^ Long-term data also suggest that surgical correction offers sustained symptomatic and survival benefits, as shown by David *et al*.^5^ Additional case series, such as that of Yamamura *et al*.^6^ have highlighted the feasibility of papillary muscle resection in selected patients.^6^

Imaging is central for preoperative recognition of such variants. Two- and three-dimensional TEE remains the gold standard for diagnosing APMI. Liu *et al*.^7^ recently introduced the ‘lip-nevus sign’ as a characteristic echocardiographic marker, improving diagnostic confidence.^7^ In our case, this sign was evident and facilitated preoperative planning. Complementary modalities such as cardiac magnetic resonance further delineate morphology and exclude fibrosis. Importantly, traditional morphometric thresholds may underestimate disease burden, particularly in women. Shiwani *et al*.^8^ reported that nearly one-quarter of genetically confirmed female patients with HCM had maximal wall thickness below the conventional ≥15 mm cutoff,^8^ highlighting the need for integrated diagnostic approaches. Although genetic testing was not performed during the index hospitalization, this was not initially considered due to the absence of a positive family history and the acute clinical circumstances. We acknowledge that comprehensive genetic evaluation is an essential component of HCM work-up, both for diagnostic refinement and for family screening, and it has been scheduled for the first outpatient follow-up visit.

Beyond diagnostics, the mitral valve apparatus itself has emerged as a key determinant of obstruction. Sherrid *et al*.^9^ demonstrated that mitral valve and subvalvular anomalies contribute significantly to pathophysiology in obstructive HCM, supporting the rationale for anatomic correction beyond septal resection alone.^9^ Extended myectomy, including resection of anomalous papillary muscles, has been performed with good results even in pediatric and young adult patients, as reported by Minakata *et al*.^10^

Beyond anatomical and surgical considerations, long-term management of arrhythmic risk remains a crucial aspect in such patients. ICD implantation was also considered in accordance with current AHA/ACC guideline recommendations for secondary prevention. These guidelines provide a class I indication after ventricular fibrillation or cardiac arrest irrespective of phenotype; however, such recommendations are not specifically tailored to rare anatomical subgroups like APMI.^11^ Observational data from large HCM cohorts demonstrate that the incidence of appropriate ICD therapies decreases significantly after successful septal myectomy, suggesting that removal of the obstructive substrate can mitigate arrhythmic risk.^12^ Moreover, conventional SCD risk calculators have not been validated in the post-myectomy setting and may overestimate risk once obstruction has been abolished.^13^ In the specific context of APMI, published series primarily focus on hemodynamic and surgical outcomes, with little systematic reporting on device therapy. Carvalho *et al*.^2^ highlighted the necessity of complete anatomical correction of papillary muscle anomalies but did not provide standardized approaches to ICD decision-making.^2,4^

In this case, the arrhythmic event was most plausibly precipitated by severe fixed LVOT obstruction from APMI, which was fully corrected by surgery. We therefore adopted a conservative strategy with interim protection by a wearable cardioverter-defibrillator^14^ and close follow-up including serial echocardiographic assessment of LVOT gradients. Should significant obstruction recur or additional risk markers emerge, definitive ICD implantation will be reconsidered. This approach aligns with reports describing temporary WCD use in the early postoperative period until the long-term arrhythmic substrate can be reassessed. Re-evaluation will include serial TTE for LVOT gradients and ambulatory rhythm monitoring; recurrent obstruction, syncope, non-sustained VT, or new high-risk markers would prompt reconsideration of definitive ICD implantation.

In conclusion, this case illustrates that APMI represents a clinically significant morphological variant of HOCM that carries a high risk of sudden cardiac arrest. Accurate diagnosis requires multimodality imaging and recognition of specific signs such as the ‘lip-nevus sign’. Optimal surgical management demands removal of all obstructive subvalvular structures to achieve durable gradient abolition. Risk stratification and ICD decision-making in this rare phenotype remain challenging, and individualized clinical judgment is mandatory.

## Conclusion

In conclusion, this case illustrates that APMI represents a clinically significant morphological variant of HOCM that carries a high risk of sudden cardiac arrest. Accurate diagnosis requires multimodality imaging and recognition of specific signs such as the ‘lip-nevus sign’. Optimal surgical management demands removal of all obstructive subvalvular structures to achieve durable gradient abolition. Risk stratification and ICD decision-making in this rare phenotype remain challenging, underscoring the importance of individualized clinical judgment and highlighting the need for multicenter registries to better define the long-term outcomes of patients with APMI-positive HCM.

## Lead author biography


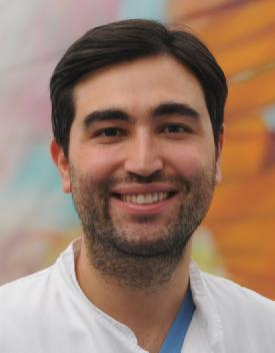


Burak Oezdemir, MD, is a cardiologist at the Department of Cardiology and Angiology, University Hospital Bergmannsheil, Ruhr University Bochum, Germany. He completed his specialist training in internal medicine and cardiology in 2024 and has a strong clinical focus on multimodal cardiac imaging, structural heart disease, and acute cardiovascular care. Dr. Oezdemir is actively involved in case-based teaching and research, with particular interest in echocardiography, HCM, and pathophysiological insights into rare cardiac variants. He is also engaged in interdisciplinary case conferences and supports advanced clinical education initiatives within his department.

## Supplementary Material

ytaf616_Supplementary_Data

## Data Availability

The data underlying this article are available within the article itself. No additional datasets were generated or analysed.
