# Supplementary figures and images for: Sudden cardiac arrest in a 47-year-old female with hypertrophic obstructive cardiomyopathy and anomalous papillary muscle insertion: a case report with advanced multimodal imaging, pathophysiological insights, and evidence-based surgical management
Source: Eur Heart J Case Rep. 2025 Nov 26;9(12):ytaf616. doi: 10.1093/ehjcr/ytaf616 (PMC12696716; doi:10.1093/ehjcr/ytaf616)

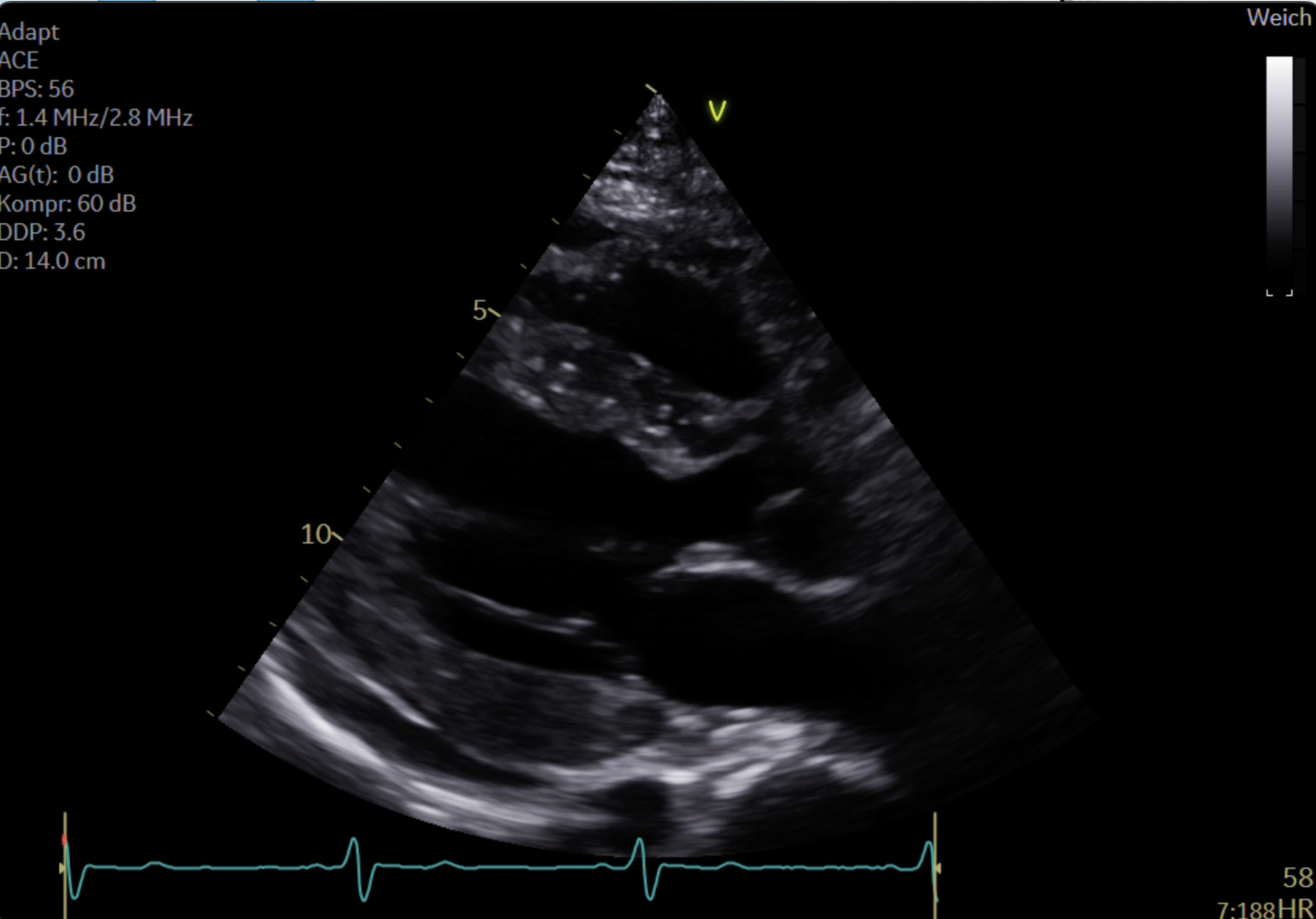

Supplement: ytaf616_Supplementary_Data [file ytaf616_supplementary_data.zip › Post-OP-Echo 1.png]

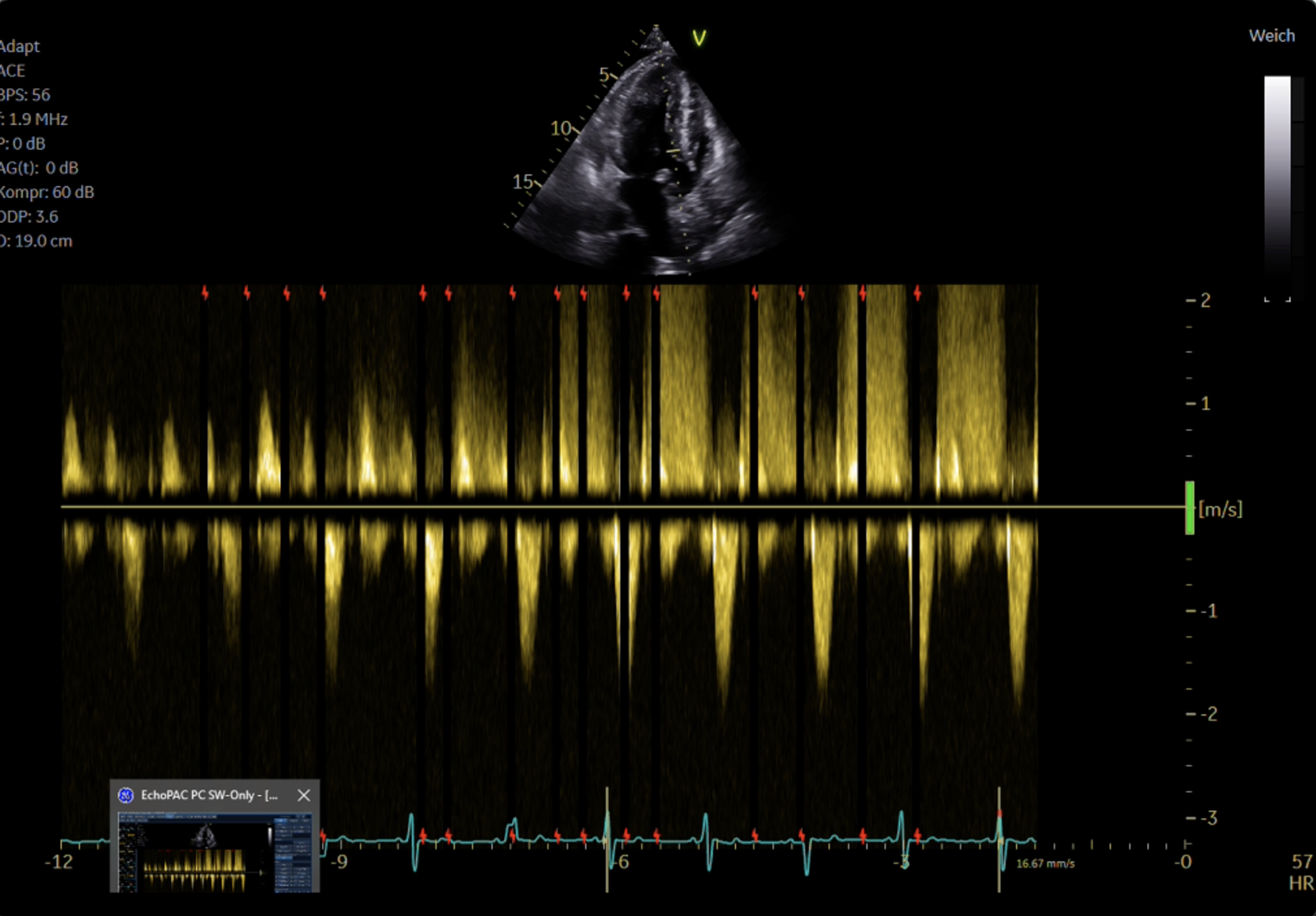

Supplement: ytaf616_Supplementary_Data [file ytaf616_supplementary_data.zip › Post-OP-Echo 2.png]

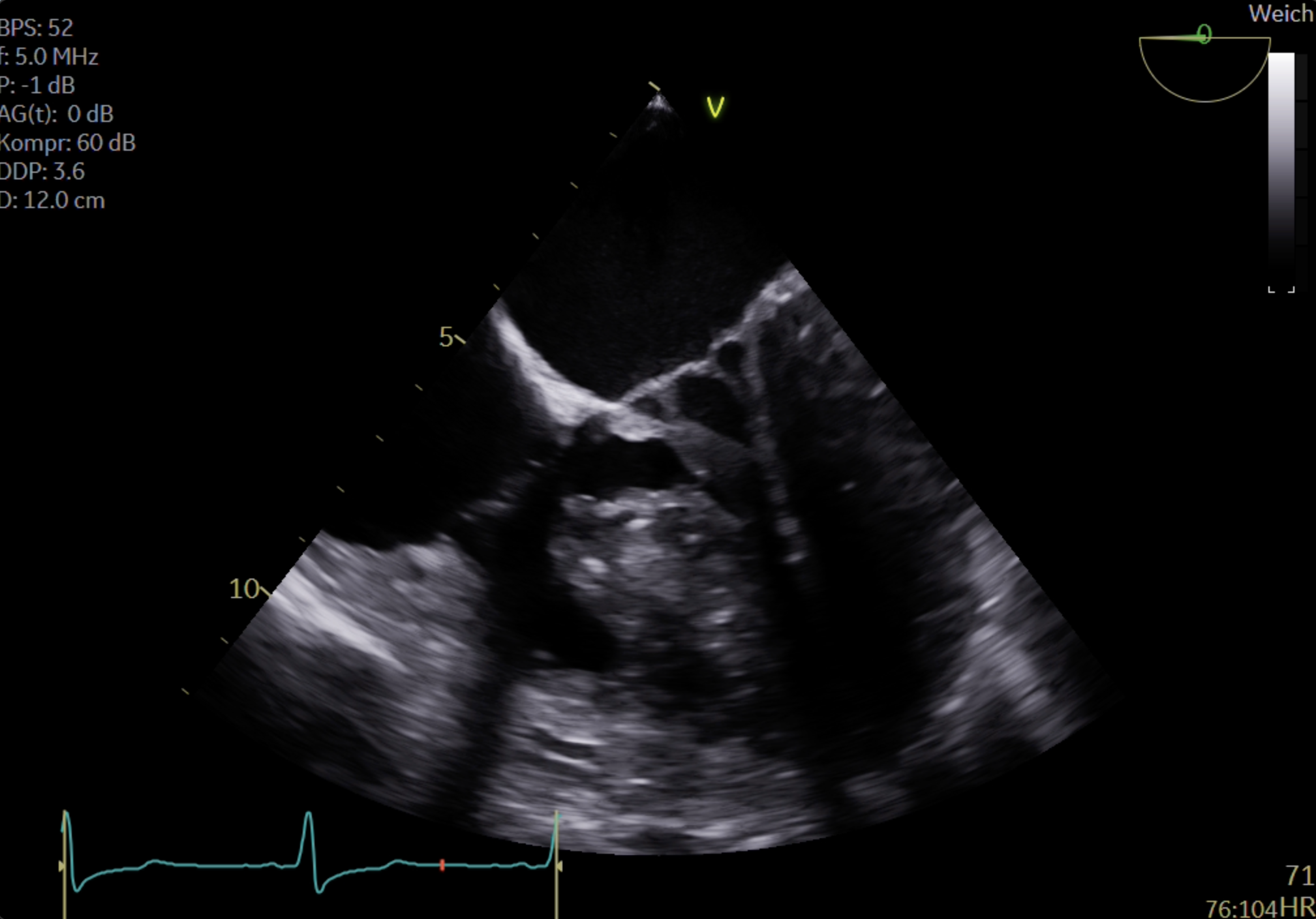

Supplement: ytaf616_Supplementary_Data [file ytaf616_supplementary_data.zip › TEE 1.png]

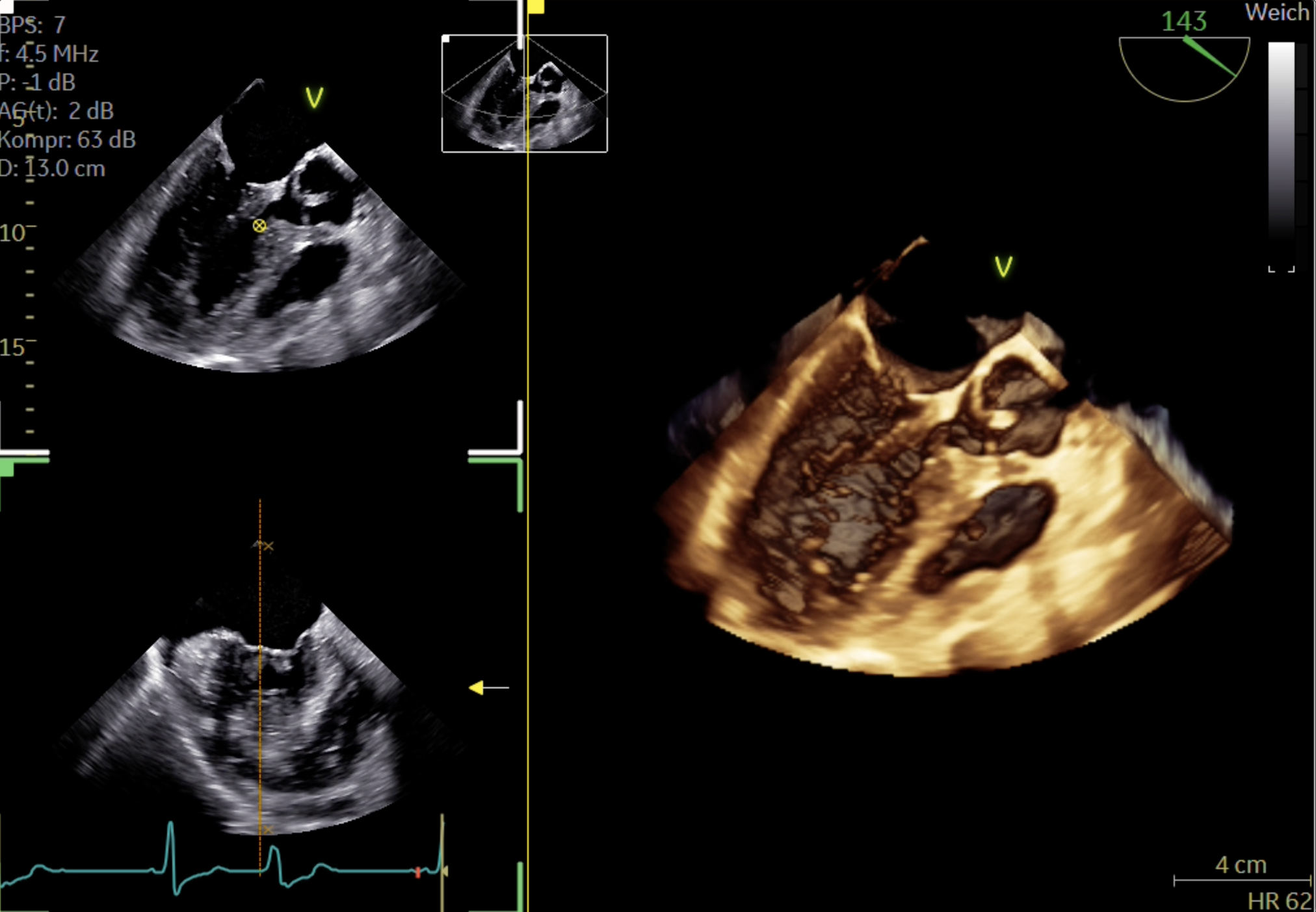

Supplement: ytaf616_Supplementary_Data [file ytaf616_supplementary_data.zip › TEE 2.png]

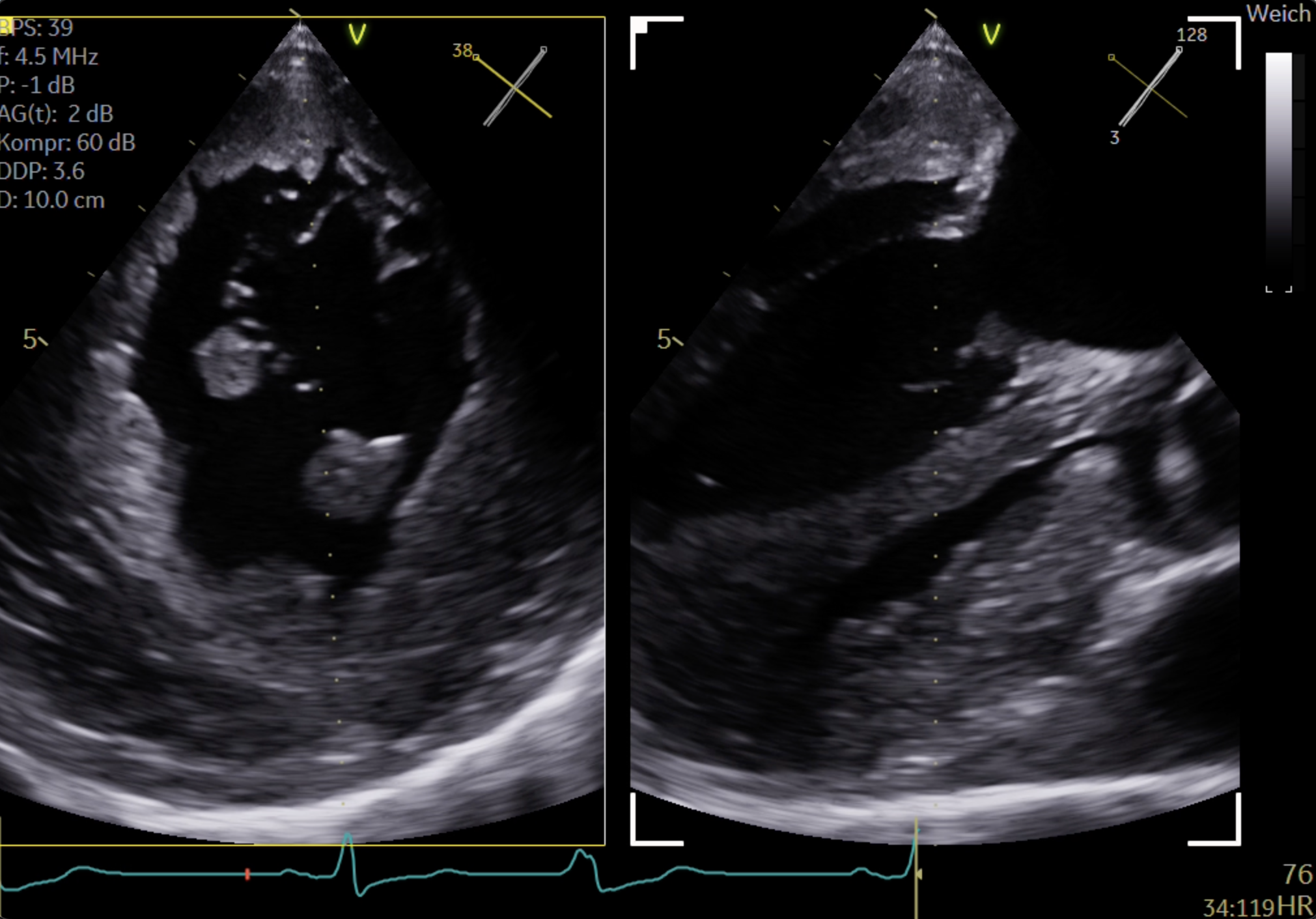

Supplement: ytaf616_Supplementary_Data [file ytaf616_supplementary_data.zip › TEE 3.png]

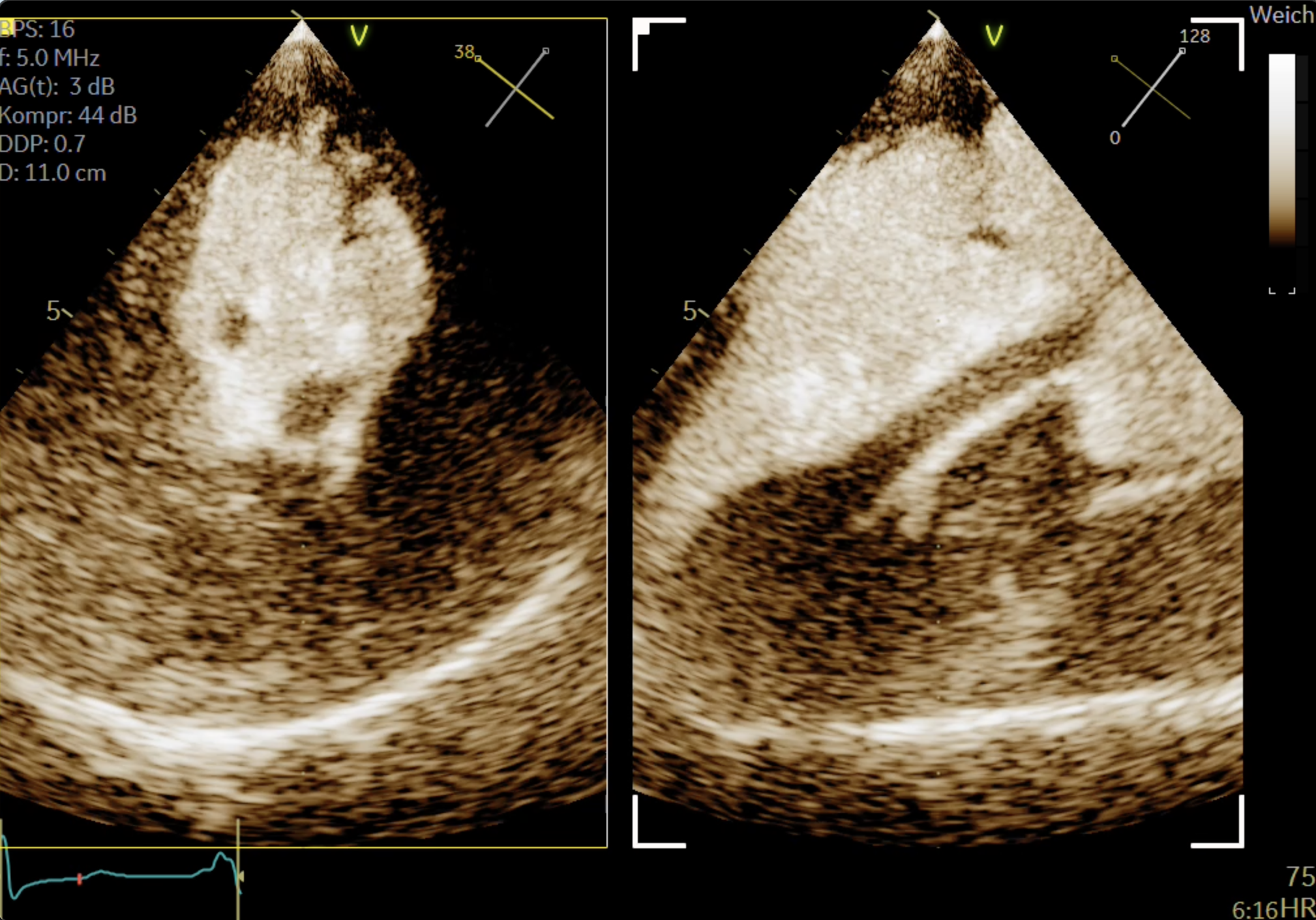

Supplement: ytaf616_Supplementary_Data [file ytaf616_supplementary_data.zip › TEE 4.png]
